# A rare mechanism of ectopy

**DOI:** 10.1007/s12471-020-01426-w

**Published:** 2020-05-11

**Authors:** D. Wesselius, J. Constandse, A. D. Hauer

**Affiliations:** 1 Haga Teaching Hospital, the Hague, The Netherlands; 2 Reinier de Graaf Ziekenhuis, Delft, The Netherlands

## Answer

**Fig. 1:**
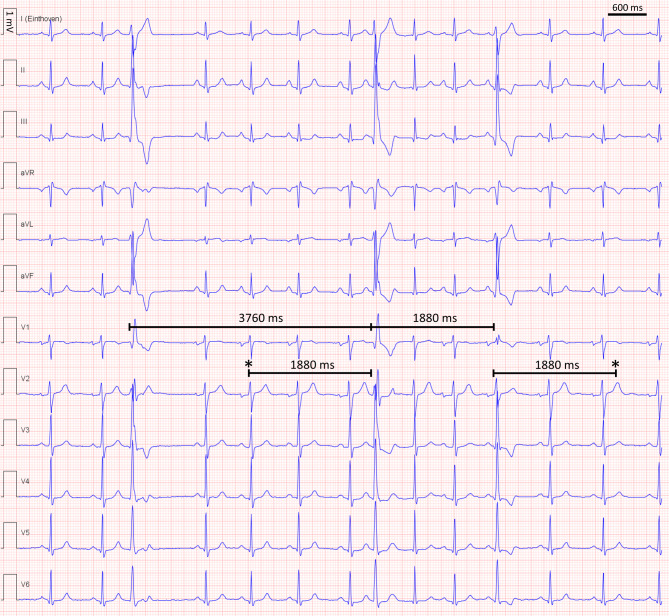
12-lead electrocardiogram showing 3 premature ventricular complexes (PVCs) with irregular coupling intervals with the preceding QRS complexes and intervals between the PVCs with a common denominator (1880 ms). *Asterisk* Illustrates expected timing of discharge of the ectopic ventricular focus which can not be propagated to the surrounding tissue because this tissue is refractory due to depolarisation by the intrinsic rhythm

The electrocardiogram (ECG) shows a sinus rhythm at 80 bpm with three broad (140 ms) QRS complexes with a right bundle branch block (RBBB) morphology and inferior axis (Fig. 1). The first two broad QRS complexes show an initial q wave in lead V1, indicating a ventricular origin of the broad QRS complexes with a morphology compatible with a focus in the left ventricular summit. RBBB aberrancy is very unlikely since the septal activation should not change during RBBB aberrancy and should therefore not lead to a q wave in lead V1. The third premature ventricular complex (PVC) is slightly shorter (120 ms) and an initial q wave is absent illustrating fusion of the PVC with the intrinsic rhythm. There are irregular coupling intervals of PVCs with the preceding QRS complexes. In addition, the interval between the first two PVCs (3760 ms) exactly doubles the interval between the last two PVCs (1880 ms) pointing to a common denominator of the intervals between the PVCs. These findings indicate an ectopic discharging focus, which is not influenced (no reset) by the intrinsic depolarisation, illustrating entrance block. However, activation originating in the ectopic focus is able to propagate to the surrounding tissue that is no longer refractory (absence of exit block). No PVC is observed at half the interval between the first and second PVC because the tissue surrounding the ectopic focus is refractory due to depolarisation by the intrinsic rhythm. This mechanism of arrhythmia is called ventricular parasystole and generally has a benign clinical course^1^. Although the patient had mild complaints of palpitations, he refused medication or catheter ablation and preferred regular follow-up of left ventricular ejection fraction.
